# Perceived causes and contributory conditions for perinatal death: perspectives of midwives, parents, and communities in Northwest Ethiopia—a qualitative study

**DOI:** 10.3389/fmed.2025.1543662

**Published:** 2025-11-10

**Authors:** Dawit Tiruneh Arega, Tesfaye Gobena, Nega Assefa, Abera Kenay Tura

**Affiliations:** 1 Department of Midwifery, College of Health Sciences, Debre Tabor University, Debre Tabor, Ethiopia; 2 School of Environmental Health Sciences, College of Health and Medical Sciences, Haramaya University, Harar, Ethiopia; 3 School of Nursing, College of Health and Medical Sciences, Haramaya University, Harar, Ethiopia; 4 Department of International Public Health, Liverpool School of Tropical Medicine, Liverpool, United Kingdom

**Keywords:** perinatal death, fetal loss, grief, social norms, cultural belief, perceived causes, contributory conditions, women empowerment

## Abstract

**Background:**

Despite many efforts to reduce perinatal death, it is common in low- and middle-income countries, including Ethiopia countries. Perinatal death has huge repercussions for parents and families, altering their self-esteem and ambitions related to parenting. Therefore, it is crucial to develop targeted and cost-effective interventions to reduce the burden of perinatal death.

**Objective:**

This study aimed to investigate how midwives, parents, and communities perceive the causes and factors contributing to perinatal death in the Lay Gayint District of Northwest Ethiopia.

**Methods:**

A phenomenological study was conducted with 14 participants (midwives, parents, and community members). Study participants were recruited through purposive sampling guided by emerging themes. A probe guide was prepared to conduct the interviews and was piloted. Two trained data collectors gathered data using in-depth, face-to-face interviews from 1 November 2022 to 30 May 2023. Participants listened to the audio records, reviewed the transcripts, and provided feedback for accuracy. Each interview was recorded and lasted between 15 and 65 min. The data were analyzed using grounded theory and an inductive theme-building approach using NVivo-14 software.

**Results:**

Three major themes emerged for the perceived causes of perinatal death: spiritual shadows, harmful traditional customs, and obstetric causes. Five major themes emerged as contributory factors: barriers to women’s empowerment, geographic and economic challenges, healthcare quality challenges, emotional turbulence, culture, and midwives as cultural mediators. This study proposed a new theory entitled “The dynamism of the culture, healthcare system, and midwifery practices in perinatal death.”

**Conclusion:**

This study emphasizes how healthcare, economic, and cultural factors interact to contribute to perinatal death, highlighting the crucial role of midwives as cultural mediators. This new theory proposes that culture, healthcare systems, and midwifery practices play a crucial role in perinatal death reduction. This study reflects the need for a multidisciplinary approach, culturally relevant interventions, and collaboration between stakeholders and cultural experts. This study provides policymakers with an understanding of how to build successful and targeted programs that consider cultural and economic costs and women’s empowerment, particularly for parents dealing with perinatal deaths.

## 1 Introduction

Perinatal death continues to be a public concern worldwide and is high in low- and middle-income countries (LMICs) (1, 2). Perinatal death has huge repercussions for parents and families, altering their self-esteem and ambitions related to parenting. Each family’s journey through this experience is individual and encompasses physical, emotional, and spiritual components (3), leading to significant psychological, financial, and long-term economic burdens (4, 5). Perinatal deaths comprise stillbirths (pregnancy loss that occurs after 7 months of gestation) and early neonatal deaths (deaths of live births within the first 7 days of life). The perinatal mortality rate is calculated as the number of perinatal deaths per 1,000 pregnancies of seven or more months’ duration (6). The exact causes of stillbirth remain unclear. However, perinatal difficulties, prolonged labor, maternal diseases, congenital abnormalities, preterm birth, hypoxia, and infection are significant causes, which are also significant causes of early newborn death (7). Many of these causes are associated with adverse social determinants of health (8).

The utilization of maternal healthcare services is limited by barriers such as distance, healthcare costs, cultural barriers, obstruction of attendance by the spouse to visit health facilities, and a lack of health awareness (9), which pushes communities toward harmful cultural and traditional practices. These harmful cultural and traditional practices ultimately contribute to perinatal death by having a significant impact on women’s reproductive practices, socioeconomic status, and pregnancy outcomes (10, 11).

A study conducted in Indonesia revealed that multicultural education implemented at an early age can improve multicultural competence (12). Another study conducted in Kenya and Uganda found considerable disparities in cultural attitudes and practices regarding infant mortality across different contexts and populations. This study examined cultural, spiritual, and supernatural beliefs as causes of stillbirth (13). The reasons for perinatal death differ by culture (14, 15). Some cultural situations may prevent parents from mourning for fear of offending God, as they perceive that God will cause additional deaths (16). A study conducted in China emphasizes the importance of self-efficacy in coping behaviors and mental health and found that news media reports affected women’s perceptions (17).

It is commonly believed that a bird’s shadow over the abdomen of a pregnant mother causes premature birth and perinatal death. This indicates how the perceived causes related to religious and traditional customs influence the community’s perception (18), causing delayed access to healthcare facilities. Women typically wait at home or opt for spiritual assistance rather than seeking medical attention because of their spiritual beliefs. A South African study explored a link between psychosocial stress experienced by midwives and neonatal deaths brought on by unfavorable working conditions, such as a lack of staff and inadequate resources (14, 15). While more than 50% of women give birth in health facilities, a large proportion still give birth at home in rural areas, often without a skilled birth attendant (19). Another study in Eastern Ethiopia revealed that low income, daily labor, a history of previous pregnancy loss, unwanted pregnancy, and a lack of antenatal care during pregnancy were contributing factors to pregnancy loss (9). Disrespectful care is a barrier to accessing care (20, 21) and promotes adherence to traditional practices. Mothers facing obstetric complications from delayed treatment can experience hostility for midwives stemming from adverse outcomes and limited resources (22). The challenging conditions in which midwives work pose risks of perinatal death.

According to the Ethiopian demographic and health survey data, the nation’s perinatal death rates have shown a gradual decrease from 52.4 to 33 in 2000 to 2016, and from 49.6 to 44 in the Amhara region (23, 24). The country faces obstacles due to health personnel shortages, financial issues, and inadequate access to healthcare. The World Health Organization (WHO) and the United Nations International Children’s Emergency Fund (UNICEF) call for coordinated efforts to eradicate preventable perinatal death, including raising awareness, decreasing stigma, assisting bereaved women, strengthening health systems, and enhancing perinatal monitoring (8). The Countdown to 2030 project seeks to enhance health, expand social opportunities, and contribute to a thriving society (25).

Despite the significant burden of perinatal death and low community awareness, few studies have explored how parents and communities perceive the causes and factors contributing to perinatal death. How do parents, communities, and midwives respond to perinatal deaths? This study aimed to investigate the perspectives of midwives, parents, and communities on how they perceive the causes and contributing factors of perinatal death in the Lay Gayint District. Furthermore, support systems and methods for dealing with challenges related to perinatal death were investigated. The findings of this study can help parents, communities, and healthcare stakeholders plan effective methods to improve maternal and child health services in the districts and regions with similar settings.

## 2 Materials and methods

### 2.1 Study settings

**Figure 1:**
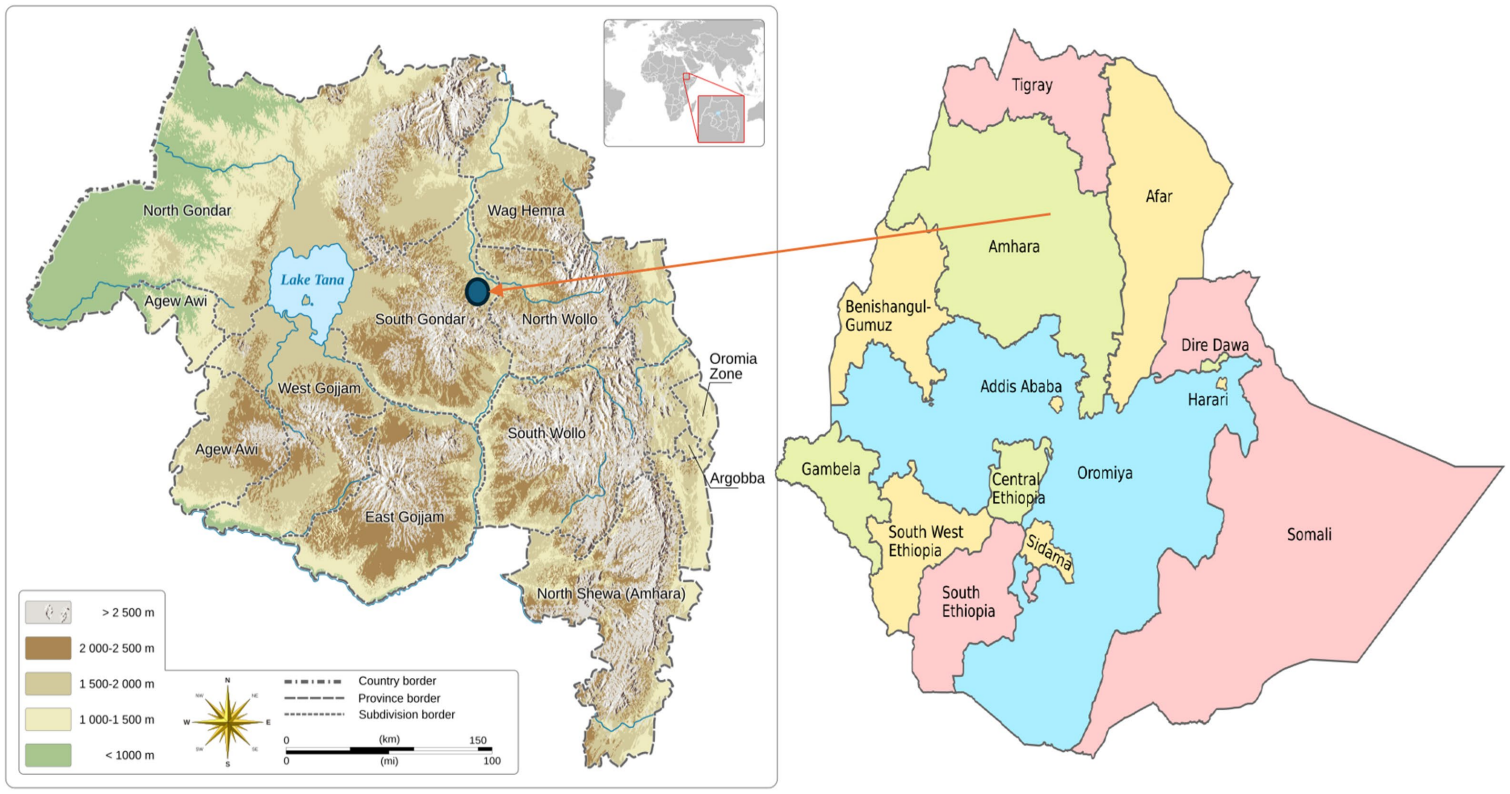
The location of Lay Gayint District in South Gondar zone, Northwest Ethiopia. Left map: reproduced from “Amhara topographic map” by Goran tek-en, licensed under CC BY-SA 4.0. Right map: reproduced from “Regions of Ethiopia EN” by Jfblanc, licensed under CC BY-SA 4.0.

Ethiopia has 14 administrative regions. Each region is divided into zones, which are further divided into woredas (districts) and kebeles (the smallest local administrative units). This study was conducted in the Amhara region of the South Gondar Zone, specifically in the Lay Gayint Woreda. Lay Gayint is a district approximately 70 kilometers east of Debre Tabor, the capital town of the South Gondar Zone. The district has 31 rural kebeles and four urban kebeles. The district’s people largely rely on agriculture, with a history of food insecurity due to tiny landholdings, degraded farmland, and irregular rainfall (26–28). The district has a population of approximately 206,000 and is served by one primary hospital, 9 health centers, and 38 health posts. Most residents are Christian (97.47%) and speak Amharic (99.84%). The regional armed conflict disrupted healthcare provision between October 2021 and July 2021. Frequent but sporadic conflicts resulted in the closure of health facilities, equipment damage, and the unavailability of valuable laboratory equipment. The study was conducted 4 months after the district’s health facilities started providing maternal healthcare services. The study lasted from 1 November 2022 to 30 May 2023 (Figure 1).

### 2.2 Study design, study population, and selection

This study used a phenomenological approach to design interview questions for understanding the lived experiences of midwives, parents, and communities. We used the Glaserian grounded theory to develop a new theory free from raw data without the use of any restrictive formula (29). We analyzed the data as they were collected, allowing for a more natural and intuitive understanding of perinatal death. The next participants were identified and interviewed based on the analyzed data, aiming to establish a data-driven theory from the perspective of parents, midwives, and community experts. To create a new theory, we took the following crucial steps: (1) use an inductive technique to create it, (2) code to find themes and connections, (3) gather more data to improve the theory, and (4) ensure that the theory makes sense and explains facts (30, 31).

The inclusion criteria for participants included the following: (1) midwives who had at least 2 years of experience working in public health facilities and had managed at least one case of perinatal death in the past 6 months; (2) parents who had experienced perinatal death within the past 6 months and had resided in the district for at least 1 year; and (3) to select community members, the study included male community members who were community leaders, religious leaders, and local health officers; knew parents who had experienced perinatal death; served as focal health officers; and had resided in the community for at least a year. We identified parents by reviewing their medical records and asking healthcare providers and local administrators. The number of study participants was determined based on the saturation of the data (32–34).

### 2.3 Data collection

We developed an interview guide by reviewing previous literature on this topic (14, 20, 35). The guide was written in English and translated into Amharic (the local language) by a multilingual expert. This was translated back into English to check its accuracy and consistency. The interview guide was piloted in a similar setting in the Farta District. The probing guide focused on the underlying causes of perinatal death, healthcare services, midwifery care, and government support. The data collection technique followed theoretical sampling, which was directed by the development of themes and insights. We used theoretical sampling, which helps us select participants based on their perspectives of a theoretical construct. Repeated data collection and analysis processes, deliberate participant selection, and continuous data collection until saturation was reached (30, 31, 36).

The Principal Investigator (PI) recruited and trained two data collectors, one man and one woman. Data collectors were familiar with cultural norms, adapting their approaches to respect participants’ backgrounds and recruiting participants relevant to the study topic. The PI explained the purpose of the study to the participants and actively engaged in the data collection process to align with the study’s objectives and rapport. Then, data were collected through in-depth, face-to-face interviews. Data collectors selected suitable interview locations to ensure that participants felt comfortable sharing their experiences and took detailed field notes after each interview. Parents and community members were interviewed in their homes, whereas midwives were interviewed in a separate room at a health facility. The Principal Investigator organized and attended all the interviews, took notes, and watched participant interactions, all of which provided valuable information for the study. The data collectors used neutral body language and eye contact while displaying empathy, allowing participants to freely express themselves. Flexibility was maintained to study unexpected but relevant themes, with open-ended responses allowing for further development. No second-round (or repeated) interviews were conducted. At the end of the interviews, all participants listened to the interview records for validation. We reassured the participants and paused the interviews when they became emotional. We checked the participants’ comfort levels during and after the interviews. Participants reviewed the transcripts of the interviews and provided feedback on their data for accuracy. Each interview was recorded and lasted between 15 and 65 min.

The interview guide consisted of the following questions:

- Could you explain what you perceive to be the cause of perinatal death?
- Could you explain how the community attempts to prevent perinatal death?
- Could you explain the barriers for a woman to access health facilities during pregnancy, delivery, and the postnatal period?
- Could you explain when your parents experienced perinatal death? How frequently?
- How do you explain midwives’ care and support to the government to improve perinatal outcomes?

### 2.4 Data quality assurance

To ensure credibility, the data collectors were trained in cultural sensitivity before data collection. The interview guide was pretested in a similar setting. The study used multiple data sources (interview transcripts, field notes, and medical cards), an experienced PI, trained data collectors, and suitable interview settings. The interviews were recorded, and nonverbal cues were noted. The participants were informed of the recording process, and their responses were authenticated upon completion of each interview. To promote transferability, we have provided thorough descriptions of our research design, methodologies, data analysis, and case descriptions to aid readers in understanding the context of the study. Dependability was assisted by our team’s competence in qualitative research and achievement of data saturation. A probe guide was prepared to conduct the interviews. This helped us to define and describe the phenomenon within certain circumstances, leading to a more accurate and nuanced understanding. For confirmability, interviews were audio-recorded, and verbatim transcripts were prepared to capture the participants’ expressions precisely. The PI compiled the respondents’ answers and conducted member checking to validate the findings.

### 2.5 Data processing and analysis

This study combined theme analysis with a grounded theory analysis, boosting the theory-building process and the overall rigor of the research (37). An eight-step grounded theory data analysis procedure was used (30). It involves (1) multiple readings, (2) open coding, (3) axial coding, (4) selective coding, (5) theory development, (6) memoing, (7) continual comparison, and (8) review and revision. The resulting theory was grounded in empirical evidence and supported by the participants’ quotes. All recordings were transcribed verbatim, and the transcripts were anonymized and imported into NVivo 14. The research team read and independently coded the data. The codes were continuously refined as themes emerged. The study team collaboratively discussed the codes and grouped the data into primary themes (38).

The data were analyzed to identify the initial themes and guide subsequent data collection and analysis. The refined themes were then integrated into a theoretical framework. This step entailed studying the interconnections between the highlighted themes and their connections to the literature and theoretical frameworks. We referred to the literature relevant to perinatal death throughout data collection and analysis. The analysis ran from thematic analysis to the development of grounded theory, which offers new and valuable insights compared with previous research. Data collection was guided by emerging analyses, with further data collection as needed. The developed theory was validated against data to ensure coherence and support.

### 2.6 Ethical considerations

The study was conducted in accordance with the Helsinki Declaration (39). This study was approved by the Institutional Health Research Ethics Review Committee of the College of Health and Medical Sciences, Haramaya University (reference number IHRERC/115/2022). Permission was obtained from the Amhara Institute of Public Health, South Gondar Zone Health Department, Lay Gayint District Health Office, and administrators of kebeles (the smallest administrative unit in Ethiopia) in the district to conduct this study. Written informed consent was obtained, and confidentiality was ensured. The PI explained the study’s aim, intended use of the data, rights of the participants, protection of their identities, secure storage of data, and benefits and potential risks of participation in this study. The participants understood that they could refuse to participate or withdraw at any time during the interview, foster trust, and enhance the quality of the collected data. Additionally, two mothers who participated in the study exhibited signs of stress and depression during fieldwork. They were linked to social support groups, visited a local health center, and received counseling after evaluation.

## 3 Results

### 3.1 The demographic characteristics of study participants

A total of 4 women and 10 men aged 26–58 years participated in this study. The study included five midwives (four men and one woman), four parents (one father and three mothers), and five community members (two religious leaders, one kebele administrator, and two focal health personnel: one nurse and one health officer). Focal health personnel, who had been working for more than 20 years in the district, were purposely selected and interviewed as community experts (Table 1).

**Table 1:** Profiles of study participants included to identify perceived causes and contributory conditions for perinatal death, Lay Gayint District, Northwest Ethiopia, November 2022 to May 2023.

Participant code	Study population	Gender	Age (years)	Marital status	Educational level	Work experience
P 1	Midwife	Male	33	Single	Degree	10 yrs.
P 2	Midwife	Male	32	Married	Degree	7 yrs.
P 3	Parent	Female (M)	47	Married	Grade 4	NA
P 4	Parent	Female (M)	40	Married	Illiterate	NA
P 5	Midwife	Male	28	Married	Diploma	4 yrs.
P 6	Midwife	Male	36	Married	Degree	9 yrs.
P 7	Community member	Male	44	Single	Diploma	NA
P 8	Community member	Male	58	Married	Grade 7	NA
P 9	Midwife	Female	26	Married	Diploma	5 yrs.
P10	Community member	Male	50	Married	Literate	NA
P 11	Parent	Male (F)	44	Married	Grade 5	NA
P 12	Parent	Female (M)	32	Married	Grade 4	NA
P 13	Community member	Male	46	Married	Diploma	20 yrs.
P 14	Community member	Male	48	Married	Degree	30 yrs.

P, participant; F, father; M, mother; NA, not applicable; yrs., years.

### 3.2 Emerged themes for perceived causes of perinatal death

Three major themes and eight subthemes emerged for the perceived causes of perinatal death: (1) spiritual shadows, (2) harmful traditional customs, and (3) obstetric causes. We found that perinatal death is negatively influenced by religious beliefs and traditional customs, leading to stigma and guilt among parents. We recognized that spirituality and tradition may also offer comfort during times of bereavement. However, traditional practices in the district prioritize cultural beliefs over evidence-based medical care, complicating pregnancy and childbirth experiences. This interplay affects maternal and newborn health outcomes. This leads to increased complications and reluctance to seek medical help. Misinterpretation of death can also negatively impact the well-being of mothers (Table 2).

**Table 2:** Major themes, subthemes, and meanings of unclear concepts.

Major themes	Subthemes	Meaning of terms/concepts
Perceived causes of perinatal death		*The perceived cause of perinatal death*, as per respondents’ perception, is the primary reason that directly leads to perinatal death, while contributory conditions are factors that may not directly cause perinatal death but contribute to the risk or severity of the situations. *“Shotelai”* represents Rhesus disease caused by blood incompatibility (Rhesus factor). *Religious beliefs:* Attributions, practices, and support systems: The belief is that perinatal death is a consequence of parental sins or transgressions. *Adherence to traditional practices:* Childbirth customs, beliefs about loss, and remedial customs. *Community knowledge gaps:* Common practices that can hinder access to healthcare. *Barriers to empowerment:* Decision-making, access to healthcare, and reproductive rights. *Cultural mediators:* Bridging cultural gaps, providing culturally sensitive care, and advocating for women’s reproductive rights.
Theme 1: Spiritual shadows	Moral misinterpretations. Cultural superstitions. Spiritual confusion.	
Theme 2: Harmful traditional customs	The heavy hand of tradition. A clash of healing practices. Pregnancy myths.	
Theme 3: Obstetric causes	Common obstetric complications. Common risk factors.	
Contributory conditions for perinatal death		
Theme 4: Barriers to empowerment	Voicelessness and power imbalance.	
Theme 5: Geographic and economic challenges	Transportation disasters. Isolation from healthcare. Economic barriers. Community knowledge gaps.	
Theme 6: The healthcare quality challenges	Unacceptable healthcare gaps. Internal conflict. Adherence to traditional practices.	
Theme 7: Emotional turbulence	Workplace strains. Societal judgments.	
Theme 8: Culture and midwives as cultural mediators	Cultural competence as connection. Trust deficits. Advocacy gaps in care. Lack of professionalism. Training and motivation deficiencies. Emotional balance in healthcare.	

#### 3.2.1 Theme 1: spiritual shadows

Perinatal death is often misattributed to spiritual beliefs, leading to harmful practices and hindering families’ ability to fully understand perinatal death. It complicates the grieving process and undermines the importance of addressing the medical realities of perinatal death, overshadowing the need for medical intervention and understanding the underlying causes.

##### 3.2.1.1 Moral misinterpretations: navigating guilt and blame

We found that perinatal death is often regarded as occurring through a moral lens, which causes emotional anguish in mourning families. This viewpoint frequently suggests that perinatal death is a divine retribution for previous crimes or moral failure. This may lead to a dread of judgment and self-blame.

“When my newborn son died, I wanted to kill myself. 'Why is God upset with me?' Why did this happen twice to me? I ask myself” (32 years old, parent).

Midwives reported that missing theological interpretations can also influence communal responses, typically by equating sympathy with judgment and preventing open discussions of grief and death. This emphasizes the significance of a compassionate perspective that distinguishes human grief from moral judgment, allowing families to mourn without fear of stigma.

##### 3.2.1.2 Cultural superstitions: the heaviness of beliefs

Community members reported that perinatal death is a deeply traumatic experience for families that is frequently perceived through spiritual and moral lenses, leading to expectations of divine punishment and guilt. Midwives reported that cultures with strong superstitious beliefs may be interpreted as penalties for real or perceived offenses by parents, particularly mothers. This can cause intense guilt and humiliation, complicate the grieving process, and isolate parents who feel criticized rather than supported. Participants underscored the necessity of open discussions on perinatal death and educated communities about the myriad medical and environmental factors that contribute to perinatal death.

“Perinatal death is connected to many things, such as not fasting during fasting seasons, adultery, or taking others' money. They believe that such actions can lead to divine retribution, often in the form of a new test” (50 years old, community member).

##### 3.2.1.3 Spiritual confusion: misguided interpretations of fate

Midwives highlighted that medical problems such as “Shotelai” [blood incompatibility] are causes of perinatal death. However, the community frequently views these issues through a spiritual lens. This results in a complex connection between medical knowledge and cultural ideas. A midwife reported that the term “loss” can undermine the acknowledgment of the neonate’s personhood and diminish its emotional impacts. Preferring backyard burials over church burials reflects low attitudes toward perinatal death.

“In our community, there is a deeply rooted tradition regarding the burial of stillborn infants and neonates who pass away before 40 days for males and 80 days for females. As religious leaders, we do not endorse this practice, nor do we teach our community to uphold it. Each child, regardless of their time with us, deserves to be honored and remembered with love and respect” (44 years old, community member).

#### 3.2.2 Theme 2: harmful traditional customs

This study highlights that harmful traditional customs refer to practices and beliefs that, while rooted in cultural heritage, negatively impact individuals or communities, particularly in terms of health, rights, and well-being. These customs can perpetuate discrimination, violence, or inequality, primarily affecting marginalized groups in the district.

##### 3.2.2.1 The heavy hand of tradition

Participants highlighted that harmful traditional practices play a pivotal role in community beliefs regarding perinatal death and prevention. This study highlights various rituals that are believed to mitigate the risks associated with childbirth and postpartum complications. This study identified several notable practices in the district, such as “Megileb.” Participants said that a close relative crossed or walked over a bleeding mother to stop postpartum bleeding. The study investigated other practices that hinder mothers from attending health facilities, such as slaughtering sheep to pacify angry spirits and firing a gun in the sky to stop postpartum bleeding at home.

“Some people perform rituals that involve creating a cloud of smoke in the house. When postpartum bleeding persists, a close relative may perform a three-time walk over the bleeding mother, known as "Megileb.” They believe that stopping these rituals can anger the spirit and lead to a problem” (44 years old, community member).

##### 3.2.2.2 A clash of healing practices

Midwives reported that the community’s reliance on traditional medicine may complicate the perceived causes of perinatal deaths in the district. They said that many pregnant women opted for traditional healers instead of seeking care from midwives. This study highlighted that traditional healers in the district lead to severe health risks for both mothers and their newborns. Participants reported that the preference of traditional healers results in women arriving at healthcare facilities with “paper–colored” conjunctiva, a term that indicates severe anemia. Participants reported that traditional healers in this district regularly utilize a traditional medicine called “Habesha medhanit,” which is composed of various herbs. Midwives reported that herbal treatments introduce harmful substances, complicating pregnancies and exacerbating risks. This study highlights the critical intersection between traditional practices and pregnancy outcomes.

##### 3.2.2.3 Pregnancy myths

The participants reported that some community members with low levels of awareness were associated with pregnancy-related conditions, such as preeclampsia, eclampsia, and hepatitis, with some causes, such as evil spirits and bird’s disease. Midwives underscored that traditional beliefs often lead to the prioritization of spiritual remedies over medical interventions. This belief can complicate perinatal outcomes in a district.

“When a pregnant woman experiences a minor disorder, people say she has been 'caught by a bird' [hepatitis]. To address this issue, traditional healers twist or cut parts under their tongues. These procedures can lead to excessive bleeding and anemia in the baby. I feel that many children are dying before receiving a treatment” (36 years old, midwife).

#### 3.2.3 Theme 3: obstetric causes

Midwives underscored that obstetric problems are risk factors for perinatal death. Participants reported that comprehensive perinatal care, early detection of problems, and effective treatment strategies are required to refine the communities’ perceptions and reduce perinatal death in the district.

##### 3.2.3.1 Common obstetric complications

Participants explained the complications for pregnant and postpartum women related to accessing vital healthcare services. They highlighted common obstetric complications that contribute to adverse perinatal outcomes in the Lay Gayint District. Midwives suggested that only a specialist clinician could diagnose the causes of perinatal death.

“Some common complications include high blood pressure, prolonged labor, anemia, and premature rupture of membranes, all of which can lead to fetal distress, birth asphyxia, and low birth weight. We often refer them to a high-level facility. Identifying a specific cause of perinatal death is challenging” (33 years old, midwife).

##### 3.2.3.2 Common risk factors

Midwives reported on the barriers that pregnant and postpartum women face in receiving critical healthcare treatments. Midwives reported that the burden of perinatal death in the Lay Gayint District is significant. This study emphasized the need for enhanced access to healthcare, community awareness, and support systems to improve perinatal outcomes.

### 3.3 Emerged themes for contributory conditions for perinatal death

Five major themes and 14 subthemes emerged for the contributory conditions for perinatal death: (1) barriers to women’s empowerment, (2) geographic and economic challenges, (3) healthcare quality challenges, (4) emotional turbulence, and (5) culture and midwives as cultural mediators (Table 2).

#### 3.3.1 Theme 4: barriers to women’s empowerment

This study emphasized that traditional family expectations and community perceptions, often prioritizing traditional values, significantly influence women’s childbearing decisions. These factors lead to feelings of voicelessness and limited autonomy. This is exacerbated by limited access to information, cultural norms, and gender biases.

##### 3.3.1.1 Voicelessness and power imbalance

This study reflects that husbands and family members have a significant influence on women’s childbearing decisions. This may result in feelings of voicelessness and limited autonomy to exercise their reproductive rights. The study also examined how family expectations and community standards typically prioritize traditional values over women’s freedom. Gender preconceptions contribute to power imbalances and women’s reproductive choices, as traditional customs favor the male gender and exclude women’s perspectives in the district.

#### 3.3.2 Theme 5: geographic and economic challenges

The study found that women experience significant challenges in accessing healthcare, especially in rural areas where services are sparse and distant. Obtaining treatment from a far distance can be difficult, especially during labor, when immediate assistance is needed. Access to crucial perinatal care may be limited, leading to an increased risk of perinatal death. Participants reported that distance, limited transportation options, and high costs can hinder women from receiving follow-up care after their initial prenatal visits. Participants reported significant barriers to timely medical care for women experiencing labor pain during workdays in distant places. They said, “Those who cannot obtain urgent assistance may have to wait for non-working days or rely on traditional birth attendants.”

“One of the most urgent health problems in our district is perinatal death. There is a lack of confidence that inadequate services offered by health centers, the issue of awareness, and the absence of sufficient resources can resolve this issue. This type of issue is common in the community. Eh…, such problems are not simple” (48 years old, community member).

##### 3.3.2.1 Limited transport options

Participants reported that limited transportation options are exacerbating the issue of distancing. They emphasized that women may not have access to reliable vehicles or public transport, making it difficult for them and their children to reach and receive appropriate care from healthcare providers. Midwives stated that the lack of transportation options led to premature death and complications, such as morbidities and lifelong disabilities.

“We are facing significant challenges in transporting mothers to the health center by ambulance. Despite a lack of ambulances, many mothers are reluctant to leave their homes, children, and husbands. Mothers and children are unfortunately losing their lives on the road” (32 years old, midwife).

Participants reported that geographical isolation in the district hinders mothers from accessing healthcare facilities. Despite restricted transportation, forcing people to stay at home during obstetric emergencies ultimately results in perinatal death. A midwife said that it is difficult to travel 5–7 h in a carriage during labor or postnatal examinations.

“Ambulances are often unavailable. If other options are available, they ask us to pay more than double. We carried out a carriage when we were sick and in labor. Our rural life is not conducive to accessing emergency care on time” (32 years old, parent).

Another participant added the following:

“When mothers come to the health center, an ambulance will often bring them. However, when they return home, they face much abuse with a baby in a public car or carriage, even during labor. This prevents them from coming to the facility” (26 years old, midwife).

##### 3.3.2.2 Economic barriers: the cost of care

Parents and community members reported that they are unable to obtain proper medical treatment. They said that they are not using the existing transport options due to financial constraints. They emphasized that travel expenses, including fuel, vehicle maintenance, and public transit, prevent women from receiving necessary medical treatment.

##### 3.3.2.3 Community knowledge gaps

Midwives emphasized the importance of breaking down cultural barriers and promoting awareness of male involvement in supporting pregnancy. They highlighted the need to reduce societal pressure and encourage family unity. Community members reported that perinatal death is solely attributed to issues concerning married women. They expressed the belief that divorce and remarriage are the only solutions for having a healthy child, rather than seeking medical advice. The midwives pointed out that this narrow perspective—that women are solely responsible for perinatal death—can lead to misdiagnosis and an inclination toward traditional healers. They also noted that cultural barriers, such as the fear of lengthy travel and the humiliation associated with seeking follow-up care, can significantly limit access to healthcare services.

“Some parents feel that one visit is sufficient, while others are reluctant to leave their homes within ten days of giving birth, and some even keep an iron rod on hand to protect themselves from negative spirits while traveling long distances during this time. Many mothers attend their first prenatal visit but may not return for follow-up check-ups because of significant distances and cultural beliefs” (32 years old, midwife).

#### 3.3.3 Theme 6: healthcare quality challenges

##### 3.3.3.1 Unacceptable healthcare gaps

This study identified factors contributing to perinatal death, including cultural influences, shortage of midwives, disrespectful treatment, inadequate medical practices, and dissatisfaction with local health facilities, leading to increased grief and hopelessness.

“They [leaders] put unqualified and fresh midwives for us. While their reception is good, they hurt mothers with excessive procedures [frequent vaginal examinations], causing uterine prolapse. They tried to treat the young girl, but she was injured. Today, the child's mother is devastated because her husband left her. The child lives in a difficult situation. She communicates with us by moving her tongue in and out while she wants food. The mother has been unable to work while caring for and feeding her child” (58 years old, community member).

Midwives reported that they are facing challenges due to a lack of essential medications and equipment, including oxytocin, vitamin K, ultrasound, fetal monitoring, and complete blood count (CBC) machines. A supply chain problem exists in this district. They said that delays in drug supply from the Ethiopian Pharmaceutical Supply Authority (EPSA) further compromised the quality of healthcare in the district. This study underscored that many women still choose home births because of gaps in service delivery, cultural influences, and high costs of private care.

##### 3.3.3.2 Regional conflicts: conflicts behind fragile healthcare settings

Participants stated that political instability in the district disrupts healthcare delivery, raises the risk of perinatal death, and impacts maternal mental health, resulting in consequences for families and communities. A midwife said that strikes, violence, and insufficient resources may impede mothers’ access to perinatal care and worsen the complications during the perinatal period.

“The closing of the health facilities due to internal conflict has had a disastrous effect on mother and child health in the district. Newborns have lost their lives as a result, and women have died of complications of PPH [postpartum hemorrhage]. The loss of a mother has profound effects on children and families” (33 years old, midwife).

##### 3.3.3.3 Adherence to traditional practices: clinging to age-old customs

Traditional birth attendants and practices may increase the risk of perinatal death by influencing women’s healthcare-seeking behaviors. Inadequate access to professional healthcare services may cause women to rely on these providers, posing risks to both mothers and children.

#### 3.3.4 Theme 7: emotional turbulence

Perinatal death can strain a marriage and even result in divorce or separation. The essential need for societal transformation is to provide healing from emotional distress. A 46-year-old community member stated that a partner who lacks understanding may end up dissolving the marriage [divorce] and blaming his wife. This strain may dissolve the relationship and family, leaving children without parents and indicating a source of pressure.

##### 3.3.4.1 Workplace strains: the unforgiving environment

Midwives reported that they are working in challenging conditions that limit their ability to deliver compassionate care. They said that resources and safety concerns limit their willingness to support their parents experiencing perinatal death. They emphasized that the lack of a safe health center due to regional armed conflicts increases stress and risk in their duties, particularly during emergency obstetrics.

“Believe it or not, we are armed with weapons. Working with weapons is challenging. As you can see, the health center has no fences and no labels [there are no signs indicating that they are health centers]. It is not straightforward to provide services in this situation. If something… [perinatal death] happens while you are attending birth at night, angry community members would attack midwives” (28 years old, midwife).

##### 3.3.4.2 Societal judgments: the burden of community perception

Participants stated that societal stigma makes parents experiencing perinatal death feel isolated and despondent. They said that some community members use stigmatizing words, “child murderers,” leading to feelings of injustice and sorrow for those accused. Parents said that stigma exacerbates their pain, leading them to seek solace in potentially deadly traditional healers.

“If we cannot give birth to a live child, we're called 'murderers,' and if we cannot get pregnant, we're called 'mules.' The community's verbal abuse is extremely irritating. I know that some women seek the help of book readers to conceive. However, I remain hopeful that God will bless me with a child” (32 years old, parent).

#### 3.3.5 Theme 8: culture and midwives as cultural mediators

This study explores how important midwives are in overcoming systemic obstacles in maternity healthcare, such as transportation, cultural viewpoints, and a lack of healthcare resources. In complex healthcare settings, midwives are essential in building trust and understanding circumstances, eventually leading to maternal service utilization.

##### 3.3.5.1 Cultural competence as a connection: building cultural bridges with midwives

Community members reported that culturally competent care is critical for helping families and midwives cope with the challenges of perinatal bereavement. They emphasized that cultural competency training leads to improved treatment and healing for parents who have experienced perinatal death by preparing families for future pregnancies and improving emotional outcomes.

##### 3.3.5.2 Trust deficits: struggles to establish rapport

This study emphasized that trust is essential for providing appropriate care, particularly during times of bereavement. Participants reported that midwives build rapport and trust with grieving parents, providing personalized care that aligns with cultural standards. This study underlined that midwives should foster a secure environment for families to communicate their concerns and fears and form emotional bonds.

##### 3.3.5.3 Advocacy gaps in healthcare: unheard voices in the healthcare system

Participants reported that midwives play an important role in advocating for healthcare reforms to reduce perinatal death. This study demonstrates how important it is for midwives to fight for a healthcare system that encourages fair access to diverse services and values, rather than only for specific families. This emphasizes that midwives can help create a more equitable healthcare system by bridging cultural concepts and practices. This study highlights the need for effective advocacy tactics in assisting all families through the grief process.

##### 3.3.5.4 Lack of professionalism: walking the tightrope of professionalism

Participants reported that there is disrespectful care given by midwives during encounters with pregnant women, those in labor, and parents experiencing perinatal death. Midwives reported that this behavior is attributed to an unfavorable work environment, heavy workloads, low motivation, and an unwillingness to seek prompt care. Some parents said that the community provides insufficient emotional support, while local authorities lack drive and oversight.

##### 3.3.5.5 Emotional balance in healthcare

This study emphasized the importance of midwives balancing empathy and professionalism when working with mourning families, fostering trust, and enhancing caregiver well-being through emotional regulation and professional boundaries.

“Mothers’ and children's health has gradually faded away. One midwife cannot handle all midwifery care. If postpartum hemorrhage and fetal distress occur together, what will I do, and what will I prioritize? When I face such a case, who is called at night? Even we do not receive recognition for our hard work” (36 years old, midwife).

### 3.4 Integration of themes

This study suggests that perinatal death is influenced by cultural, economic, and healthcare factors. We constructed three statements based on the previously indicated essential themes. These include:

1. Perceived causes of perinatal death are influenced by culture.
2. Contributing factors to the healthcare system exacerbate perceived causes of perinatal death.
3. Midwives can reduce the contributing factors and refine perceived causes of perinatal deaths, serving as cultural mediators.

**Figure 2:**
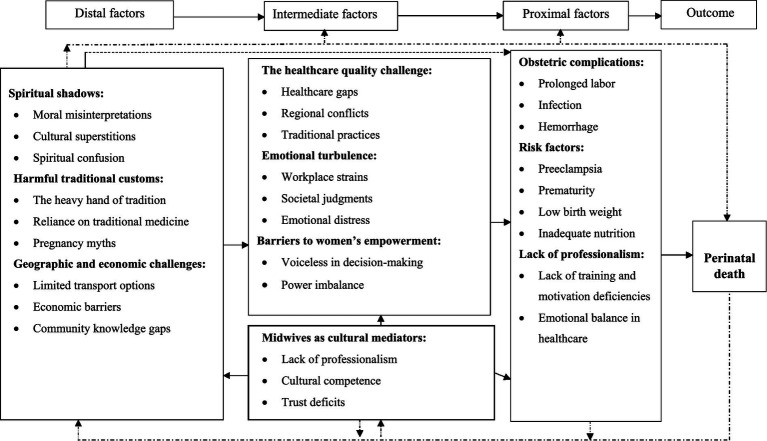
Theoretical framework of culture, healthcare, and midwifery dynamics in perinatal loss.

These insights led to the development of the new theory, “*The Dynamism of Culture, Healthcare, and Midwifery Practice in Perinatal Death*.” The theory emphasizes the importance of culturally competent care and the role of midwives in the healthcare system (Figure 2).

## 4 Discussion

### 4.1 Main discussion

Perinatal death is a significant public concern in low- and middle-income countries (1), including Ethiopia. It is a broad and complex phenomenon in areas with low educational levels, limited resources, and political instability (40). This study examines the perceived causes and contributing factors of perinatal death in the Lay Gayint District of Northwest Ethiopia, covering eight major themes.

Perceived causes include spiritual shadows, harmful traditional customs, and obstetric causes. The contributing factors include barriers to women’s empowerment, geographic and economic challenges, healthcare quality challenges, emotional turbulence, and culture and midwives as cultural mediators. Midwives can help close these gaps and improve healthcare access. Cultural beliefs significantly influence emotional responses and healthcare decisions. These hypotheses led to a new theoretical framework called “*The dynamism of culture, healthcare, and midwifery practices in perinatal death.*” Perinatal death in the Lay Gayint District is frequently attributed to divine anger, which causes guilt, humiliation, and social disgrace (41). Traditional customs, such as backyard burials, frequently rely on spiritual rather than obstetric reasons (42). The community’s preference for backyard graves emphasizes the need for increased recognition of perinatal death within cultural standards (43), as well as greater recognition and support for parents facing perinatal death (44, 45). The study also found geographic, economic, and cultural barriers to healthcare services that cause prenatal treatment delays (46). Inadequate transportation, care discontinuity, political instability, and isolated habitation exacerbated the lack of access to healthcare. Thus, communities must recognize and challenge these beliefs in creating a more supportive environment. Shortages of midwives, as well as medical supplies and equipment, compromise treatment quality and increase the risk of perinatal death (26, 46). Despite the district’s transportation problems, reliance on traditional ambulances for treatment, home delivery, and considerable care discontinuity are caused by remote areas and a lack of decision-making autonomy (47).

This study highlights the importance of culturally competent training for midwives in the Lay Gayint District in order to understand how to deal with perinatal death (48). A study in Indonesia underscores that cultural education must be a concern and implemented at an early stage to increase awareness and tolerance in religion, ethnicity, culture, and customs (12). Incorporating culture into medical education is important to comprehend and acknowledge the varied cultural perspectives on bereavement. Midwives must get short- and long-term training. This makes it possible for them to assist bereaved families more successfully. To provide individualized care that is in line with their cultural beliefs, midwives should establish a comfortable environment where parents can speak aloud about their worries and anxieties (49, 50). To create a more equitable healthcare system, midwives should advocate for structural changes that minimize barriers to obstetric and midwifery care (51). This study emphasized the importance of culturally safe prenatal support groups as well as culturally sensitive midwifery training to effectively manage emotions and care for mourning families (14, 35). This study stresses the need for midwives to understand and respect different cultural customs and beliefs.

This study found that a lack of women’s empowerment related to decision-making autonomy led to high care discontinuity. Most women received maternity care with the willingness of their husbands and families. In addition to healthcare system gaps, distance, lack of awareness, and equipment contribute to care discontinuity. Midwives’ jobs are vital for perinatal death reduction, but they are often dissatisfied due to their dissatisfaction with pay and working conditions, leading to career changes and causing stress for them (14, 16, 52). This study emphasized the need to address midwives’ concerns, particularly during and after conflict, in situations with low resources.

In Jimma, Ethiopia, nearly half of the midwives planned to leave their current facility, and more than half expressed dissatisfaction with their jobs (53). Another study conducted in Addis Ababa, Ethiopia, revealed that midwives were not satisfied with their salary, incentives, and career development (54) but they were most satisfied with their relationships with coworkers and the care they provided to clients (54, 55). It is crucial to invest in evidence-based maternity care in low-income countries despite transportation, distance, and cultural barriers (56).

This study found that open communication, active listening, empathy, and respectfulness would improve healthcare quality, especially for families living with perinatal loss.

This study emphasized the importance of midwifery care, community knowledge, and treatment quality in reducing perinatal death. Perinatal death impacts households and the national economy, and this study informs Ethiopia’s progress toward attaining sustainable goals by 2030. This study identified that low-income families may be unable to afford costs related to emergency care and discovered that the fundamental causes of perinatal death are poorly understood scientifically. This study also found that communities do not understand the reasons for perinatal death. Thus, midwives are expected to provide compassionate care to overcome cultural barriers. In addition, the study was conducted in the aftermath of Ethiopia’s internal conflicts, which have had a significant impact on access to care due to health facility closures and a lack of ambulances and other modes of transportation, causing maternal stress and exacerbating the scarcity of resources, equipment, and drugs in health facilities. These ongoing conflicts primarily affect silent mothers, their children, and their families. Therefore, policymakers can improve perinatal outcomes in the district by addressing factors that influence healthcare systems, midwives’ attitudes, and community awareness. This study emphasized the difficulties that disadvantaged people have in receiving obstetric treatment, such as restricted access, financial restraints, and cultural hurdles, along with the importance of resolving these challenges through a comprehensive plan that includes midwives. This study reflects the need for a holistic approach to support families dealing with perinatal death, focusing on cultural and economic factors. This study’s findings suggested that greater emphasis should be placed on preventing and reducing perinatal death, as well as better supporting parents in understanding it after it occurs. The study suggests working sensitively with spiritual leaders to develop culturally and spiritually acceptable understandings of fetal death, which necessitates timely attendance in emergency care. Parents and community members reported that mothers are refusing vaginal examinations during childbirth and other procedures due to cultural influences, overcrowded healthcare facilities, and a lack of privacy. This study identified the role of midwives as cultural mediators in which sexual matters are sensitive to discussing and performing certain procedures. Maintaining many mothers in a single room and corridor during labor and the postnatal period may contradict their culture. This study also recommends incorporating cultural competence training into midwives’ education and developing comprehensive support systems. This study advocates for a multifaceted approach that prioritizes cultural sensitivity and economic considerations in healthcare.

Working collaboratively, the Ministry of Health, Regional Health Bureaus, and Midwifery Associations can create a desirable working environment, address transportation challenges and traditional practices, and increase district service consumption. Midwifery models of care involve educated, licensed, and regulated midwives providing high-quality care to women and newborns from pre-pregnancy to postnatal stages. This approach promotes a person-centered approach, values the woman-midwife partnership, and optimizes physiological, biological, psychological, social, and cultural processes. Midwives collaborate within networks of care, ensuring personalized care tailored to each woman’s and newborn’s health needs (57).

We recommend that the Ethiopian Ministry of Health enhance compassionate and respectful care by providing training for the health workforce on compassionate and respectful care, having a favorable working environment, and incorporating pre-service education, advocacy, and system strengthening. We suggest that there is a huge need for investment in antenatal education of parents regarding warning signs of obstetric adverse events, improving the healthcare system, improving infrastructure and transport options, the availability of ambulances in rural settings, and midwives’ training, supervision, support, and remuneration.

This study recommends additional research, policy amendments, and increased funding for maternity care, coordination, emotional support, awareness campaigns, and cultural competency training for midwives and parents. The study used a qualitative approach with thorough data collection procedures to gain insight into the perspectives of midwives, parents, and community experts to explore perceived causes and contributing conditions to perinatal death in the district. The Consolidated Criteria for Reporting Qualitative Research (COREQ) checklist for comprehensive reporting of qualitative studies (58) was followed in this study.

### 4.2 Strengths and limitations of the study

A more detailed understanding of participants’ experiences is possible with qualitative approaches, which yield rich data that quantitative studies can overlook. These findings are more credible because of the study team’s experience. A thorough examination of perinatal death reveals viewpoints on the body of material already in existence and guides future investigations. Collecting data from three different groups was a major strength of this study. This allows other researchers to compare different perspectives and achieve a comprehensive and multifaceted understanding of the phenomenon. Although perinatal death is inextricably linked to the mothers’ bodies, as well as their psychosocial and cultural backgrounds, only four male participants were interviewed (Table 1). We interviewed more men for this study because of the influence of culture on interviewing voiceless teenagers and mothers. In addition, the district had only a few female midwives, some due to internal turmoil, instability, and topography. Given their significance, these factors may have introduced bias into this study. This study is limited by the lack of a gender balance among participants, which reflects barriers to engaging women in research studies in this region.

These underrepresentation’s may affect the generalizability of our findings. The study’s integrity may be impacted by various biases, such as varying interpretations of the results and subjective judgment of data saturation. Therefore, the findings and conclusions should be carefully interpreted in the district.

## 5 Conclusion

The complex interactions between healthcare, economic, and cultural factors that lead to perinatal death are emphasized in this study. Perceived causes include spiritual shadows, harmful traditional customs, and obstetric causes. The contributing factors include barriers to women’s empowerment, geographic and economic challenges, healthcare quality challenges, emotional turbulence, and culture and midwives as cultural mediators. The study develops a new theory entitled “*The dynamism of the culture, healthcare system, and midwifery practices in perinatal death*.” This study reflects a multifaceted approach that prioritizes cultural sensitivity and economic considerations in healthcare to improve perinatal outcomes, specifically for parents affected by perinatal death. The study recommends incorporating cultural competence training into midwives’ education and developing comprehensive support systems that encompass health resources, community outreach initiatives, and educational programs.

## Data Availability

The original contributions presented in the study are included in the article/supplementary material, further inquiries can be directed to the corresponding author.
